# DNA Polymerase α (*swi7*) and the Flap Endonuclease Fen1 (*rad2*) Act Together in the S-Phase Alkylation Damage Response in *S. pombe*

**DOI:** 10.1371/journal.pone.0047091

**Published:** 2012-10-11

**Authors:** Milana Koulintchenko, Sonya Vengrova, Trevor Eydmann, Prakash Arumugam, Jacob Z. Dalgaard

**Affiliations:** 1 Division of Biomedical Cell Biology, Warwick Medical School, Gibbet Hill Campus, University of Warwick, Coventry, United Kingdom; 2 School of Life Sciences, Gibbet Hill Campus, University of Warwick, Coventry, United Kingdom; University of Massachusetts Medical School, United States of America

## Abstract

Polymerase α is an essential enzyme mainly mediating Okazaki fragment synthesis during lagging strand replication. A specific point mutation in *Schizosaccharomyces pombe* polymerase α named *swi7-1*, abolishes imprinting required for mating-type switching. Here we investigate whether this mutation confers any genome-wide defects. We show that the *swi7-1* mutation renders cells hypersensitive to the DNA damaging agents methyl methansulfonate (MMS), hydroxyurea (HU) and UV and incapacitates activation of the intra-S checkpoint in response to DNA damage. In addition we show that, in the *swi7-1* background, cells are characterized by an elevated level of repair foci and recombination, indicative of increased genetic instability. Furthermore, we detect novel Swi1-, -Swi3- and Pol α- dependent alkylation damage repair intermediates with mobility on 2D-gel that suggests presence of single-stranded regions. Genetic interaction studies showed that the flap endonuclease Fen1 works in the same pathway as Pol α in terms of alkylation damage response. Fen1 was also required for formation of alkylation- damage specific repair intermediates. We propose a model to explain how Pol α, Swi1, Swi3 and Fen1 might act together to detect and repair alkylation damage during S-phase.

## Introduction

DNA polymerase alpha (Pol α) is an essential replicative DNA polymerase, which is conserved amongst all eukaryotes. The Pol α holoenzyme consists of four subunits: a catalytic subunit, a regulatory beta-subunit and two primase subunits. The primase entity of DNA Pol α synthesises a ∼10 nucleotide long RNA primer at the origins of replication and at the start of each Okazaki fragment during lagging strand synthesis. This RNA primer is extended, with low fidelity, by the catalytic subunit of Pol α holoenzyme by 30–40 nucleotides, and the synthesis is completed by a highly processive and high-fidelity DNA polymerases δ or ε. During maturation of Okazaki fragments, the part of each fragment, synthesized by Pol α, is displaced by processive DNA synthesis and removed by the flap endonuclease Fen1 and the DNA helicase and endonuclease Dna2.

The catalytic subunit of Pol α contains 6 conserved domains (designated I to VI in the order of the most conserved to least conserved), the first (I) of which is important for interaction with metal ions, Mg^2+^ and Mn^2+^, and the second (II) – for dNTP binding [1]. While regions I, II and III are present in all eukaryotic and prokaryotic polymerases, the region V is present only in all eukaryotic polymerases, except those encoded by maize S1 and yeast pGKL1 plasmids [1]. Interestingly, a single amino acid substitution G372E in Pol α catalytic subunit in *Schizosaccharomyces pombe* affects imprinting at the mating-type locus without abolishing its essential catalytic activity [2].

The fission yeast exhibits two opposite mating types “+” and “−”. Cells can switch their mating-types by reprogramming their genomic DNA (reviewed by [3]). The mating-type of *S. pombe* is determined by the *mat1* locus located on chromosome II. Mating-type switching occurs when the DNA of the actively expressed cassette at *mat1* is replaced with a cassette that contains the opposite mating-type gene sequence [4]. The gene conversion event resulting in switching of mating type is initiated by an imprint at the *mat1* locus that is formed during S-phase of the cell cycle [5], [6]. The imprint has been characterized as two ribonucleotides, incorporated into the DNA duplex at a specific position, and it is formed in the strand, which is synthesized as lagging during replication of *mat1* [7], [8]. Formation of the imprint depends on pausing of the replication fork at the *mat1* pause site 1 (*MPS1*) mediated by Swi1 and Swi3 [9]. The *swi1*, *swi3* and *swi7-1* mutants were identified together in a screen for the factors affecting mating-type switching [10]. In *swi1* and *swi3* mutants, both replication pausing and imprinting at *mat1* are abolished, while the *swi7-1* mutation only affects imprinting. Thus, Swi7/Pol α acts downstream of Swi1 and Swi3 in the imprinting pathway [9]. Swi1 and Swi3 were subsequently shown to have genome-wide functions. They were characterized as parts of a Replication Fork Progression complex [11], [12]. These factors travel with the replication fork and are involved in response to alkylation damage and intra S-phase checkpoint signalling [11], [12], [13]. In addition to their function at the *mat1* pause site *MPS1*, Swi1 and Swi3 were also shown to act at replication termination site *RTS1* and at the ribosomal DNA (rDNA) barriers [9], [14].

Here we investigated whether the *swi7-1* mutation confers any genome wide effects outside the mating-type region. We show that the *swi7-1* mutation causes general genetic instability and hypersensitivity to DNA damaging agents, in particular to alkylation damage. Specifically we observe Swi1-, Swi3-, Pol α and Fen1 dependent alkylation damage repair intermediates. Furthermore, we show that Fen1 and Pol α act in the same pathway with regards to alkylation damage repair. We propose how Swi1, Swi3, Pol α and Fen1 could act together in DNA repair at replication forks stalled at damaged bases.

## Results

### S*wi7* mutation increases sensitivity to DNA damaging agents

To investigate the role of Pol α in the DNA damage response, we measured the sensitivity of *swi7* mutant cells to MMS, HU and UV. The alkylating agent MMS leads to formation of three types of DNA damage depending on the nature of CH_3_ attachment at the bases [15]. In 80–90% of cases, MMS treatment results in alkylation of purines (N^7^-guanine and N^3^-adenine), which are repaired by base excision repair (BER; [16]). The N^7^-adenine and N^3^-guanine make up to 1% of the damage and the O^6^-guanine (MeG) up to 0.3%. MeG is the most potent damage induced by MMS, as this modification cannot be repaired by BER but has to be removed directly. Alkylation damage has been shown to slow S-phase progression by FACS analysis [13], [17]. Some alkylation-damaged bases including 3-methyladenine and 3-metylguanine act as barriers for DNA replication [18], [19].

**Figure 1:**
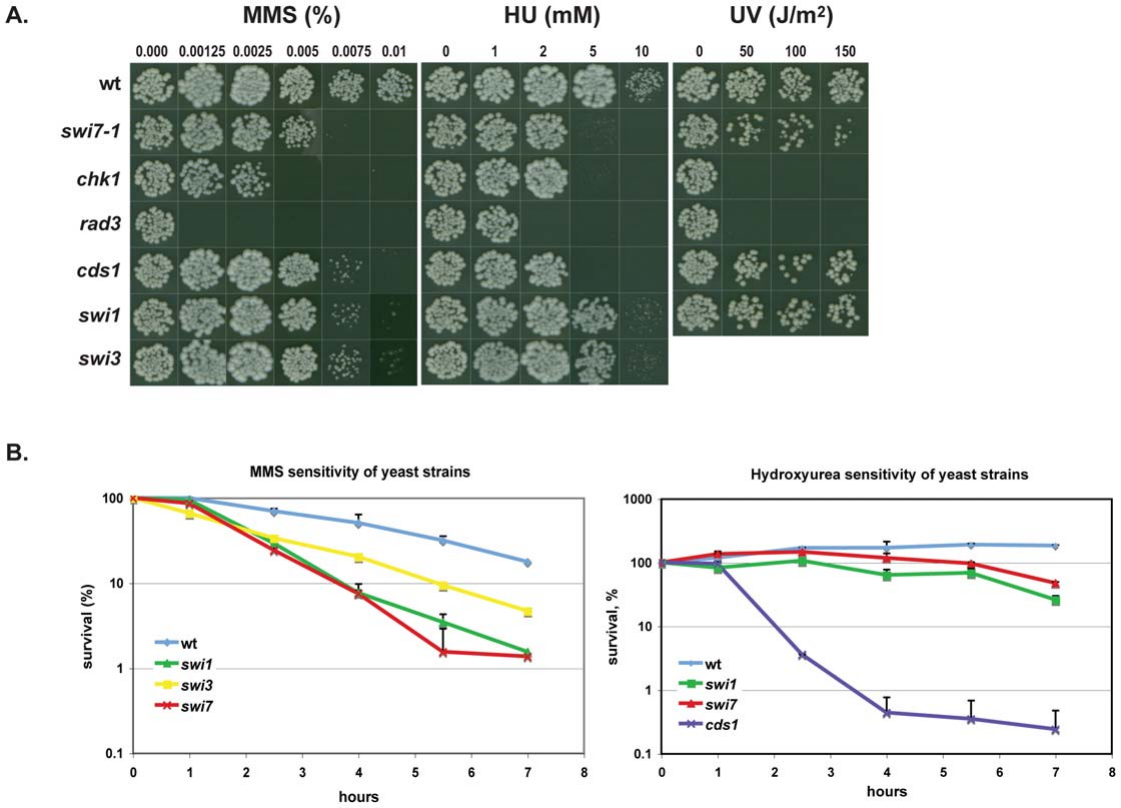
*S. pombe swi7-1* mutant is sensitive to DNA damaging agents. **A.** Drop-assay analysis. Drops containing approximately 100 cells from mutant and wild type strains were placed on medium, containing methyl methanesulfonate (MMS) or hydrohyurea (HU) at concentrations given, or were exposed to UV at the given intensities. The plates were incubated at 33° until colonies were formed. **B.** Survival of yeast cells exposed to MMS or HU. Logarithmically growing cultures were treated with either 0.03% MMS or 12 mM of HU. Samples were taken at indicated time points, and dilution series of cells for each culture were plated on YEA media and incubated at 33°C until colonies were formed. Strains shown in panel A and B are *wt* (JZ60), *swi7-1* (JZ468), *chk1* (JZ473), *rad3* (JZ474), *cds1* (JZ475), *swi1* (E111), *swi3* (E146).

Test of survival following chronic exposure to MMS was performed using the drop-assay approach. Results indicated that the *swi7-1* mutant was hypersensitive to MMS compared to wild type strain (Figure 1A). Its sensitivity was similar to that of *swi1*, *swi3* and *cds1* mutant strains (cell lethality was observed at 0.005–0.0075% MMS) but not as high as that of *chk1* and *rad3* mutant cells [13]. Test of survival after limited exposure to MMS also indicated that the *swi7-1* mutant was hypersensitive to alkylation damage (Figure 1B). This hypersensitivity was comparable to that of the *swi1* mutant strain but slightly more than the *swi3* mutant strain.

Hydroxyurea (HU) is a DNA replication inhibitor that represses both the elongation and initiation phases of replication and triggers the intra-S phase checkpoint. Hydroxyurea affects DNA synthesis by reversibly inhibiting ribonucleotide reductase (RNR), preventing the reduction of ribonucleotides to deoxyribonucleotides (deoxynucleoside triphosphates [dNTPs] thereby depleting the dNTP pool at replication forks [20]. HU slows down or inhibits S-phase progression and can compromise genetic integrity by increasing the rate of recombination. The *swi7-1* mutant cells were hypersensitive to HU (4 mM) (Figure 1A). The HU sensitivity of *swi7-1* cells was comparable to that of checkpoint mutants *cds1* and *chk1*, but slightly elevated compared to *swi1* and *swi3* mutants. However in the short-term survival test (Figure 1B) there was no difference between wild type and *swi7-1* sensitivities to HU. This suggests that *swi7-1* mutant cells lose viability after prolonged exposure to HU (more than 5–6 hours).

The principal forms for DNA damage induced by UVB and UVA are cyclobutane pyrimidine dimers and 6,4-pyrimidine-pyrimidone photoproducts, while UVC mostly forms dipyrimidines that contain 5-methylcytosine [21]. These photoproducts are mutagenic and block DNA replication by the replicative DNA polymerases [22]. The *swi7-1* mutant cells were only mildly sensitive to UV irradiation (Figure 1A). Cell growth began to decline slightly only when yeast cells were subjected to a 150 J/m^2^ dose of UV. These results indicate that the *swi7-1* mutation affects the ability of Pol α to respond to alkylation and HU–induced DNA damage.

### *swi7* mutation affects the intra-S phase checkpoint

**Figure 2:**
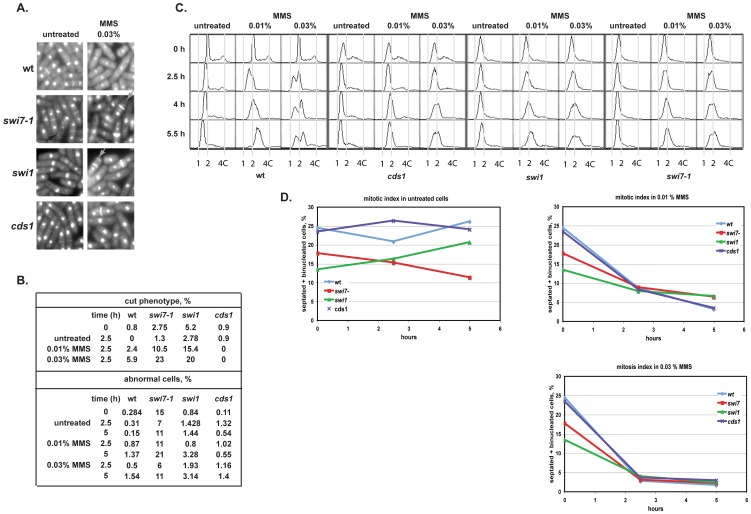
*swi7-1* mutant might have a role in S-phase but not in mitotic checkpoint. **A.** Images of the *wt* (JZ60), *swi1* (E111), *swi7-1* (JZ468) and *cds1* (JZ475) cells stained with DAPI and Calcofluor were taken at 2.5 h and 5 h time point for both the untreated and MMS treated cultures. Gray arrows indicate cells with the cut phenotype. Five hundred or more cells were analyzed for each strain. **B.** Quantification of the percentage of cells displaying the “cut” phenotype and abnormal nuclei for the indicated strains, for both treated and untreated cultures. **C.** The *swi7-1* mutant shows defect in S-phase progression in response to MMS damage. Asynchronous cultures of *wt* (JZ60), *cds1* (JZ475), *swi1* (E111) and *swi7-1* (JZ468) strains were incubated in absence or presence of 0.01% or 0.03% MMS. Cell samples were taken for flow cytometry analysis at indicated time points. **D.** Mitosis index of *wt*, *cds1*, *swi1* and *swi7-1* cells. Changes in the percentage of binucleated and septated cells were determined as a function of time without and after the addition of 0.01% or 0.03% MMS.

DNA damage activates checkpoint mechanisms, which prevent entry into mitosis until DNA is repaired. However if the intra-S checkpoint is defective then cells will enter mitosis despite the presence of damaged DNA resulting in mitotic chromosome segregation defects and cell death. Typically such cells will manifest a “cut” (for “cell untimely torn”) phenotype which consists of a septated cell with abnormally segregated DNA easily detected by DAPI staining [23]. Wild-type and *swi7-1* mutant cells were grown to log phase and stained with DAPI for visualization of nuclei. The *swi7-1* showed significantly increased amount of cells with the ‘cut phenotype’ even in the absence of MMS treatment (Figure 2A and B) and this was further increased by prolonged (2.5 and 5 hours) treatment with 0.01% and 0.03% MMS. Also, more *swi7-1* cells with abnormal nuclei were observed when cell were exposed to 0.01% MMS, but not when exposed to 0.03% MMS.

Since we detected a high level of cells with the “cut” phenotype for both MMS exposed and unexposed *swi7-1* cells, we investigated whether the *swi7-1* mutant cells had any checkpoint defects. First, we examined the effect of *swi7-1* on the intra-S phase checkpoint response. Activation of the intra-S phase checkpoint can be determined by exposing logarithmically growing cultures to MMS and analysing the DNA content of the cells by flow cytometry [17], [24]. In fission yeast, during exponential growth, G2 phase occupies approximately 70% of the cell cycle. When DNA synthesis is arrested due to activation of the intra-S phase checkpoint, there will be two populations with 1C (cells arrested in S) and 2C DNA content (cells arrested in G2). While wild type cells demonstrated a slowing down of replication under alkylation stress, and the appearance of two peaks in the FACS profile, the *swi7-1* mutant cells did not (Figure 2C). The same effect was observed in *swi1* mutant cells and to a greater extent in the *cds1* mutant, which are known to be defective in the intra-S phase checkpoint. This shows that Pol α is required for intra-S phase checkpoint activation following DNA damage.

We then investigated whether the *swi7-1* mutation affected the mitotic checkpoint. Since formation of a septum is dependent on prior mitosis, we measured the percentage of cells with two nuclei (binucleated cells) and with a septum as an indication of passage through mitosis. Nuclei and septation can be visualized in fixed cells by staining with DAPI (DNA binding dye), and Calcofluor (septum dye), followed by UV illumination. DAPI and Calcofluor staining of cells prepared from asynchronous wild type, *cds1*, *swi1* and *swi7-1* strains cultures, treated by MMS, revealed that in all tested strains, the proportion of binucleated and septated cells was significantly reduced within 2–3 h of exposure to 0.01 and 0.03% MMS (Figure 2D), indicating that both *swi7-1* and *swi1* strains displayed normal mitotic arrest. The fact there is an intact G2-M checkpoint response in the *swi7-1* strain is not in conflict with the observation of a cut phenotype. Indeed similar observations have been made in *sap1* mutant cells where the presence of damaged DNA that cannot be fully repaired affects faithful chromosome segregation resulting in the appearance of the “cut” phenotype (Figure 2A; [25]). Thus, the *swi7* cells arrest in mitosis in response to DNA damage, but might show the “cut” phenotype due to accumulation of DNA damage.

### Detection of alkylation damage repair intermediates in *S. pombe*

**Figure 3:**
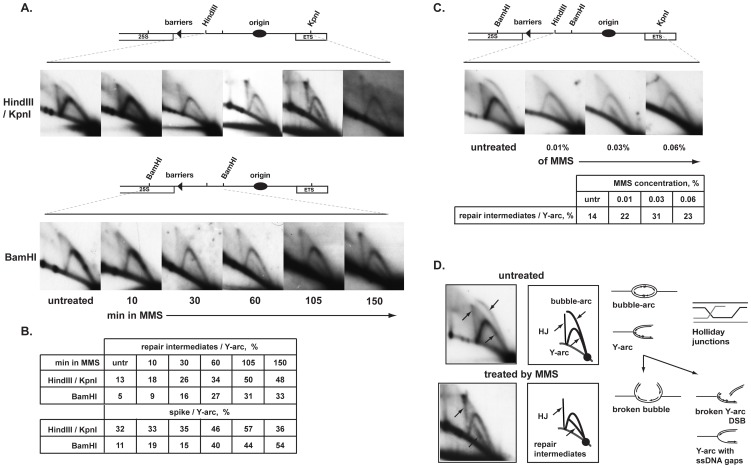
Analysis of replication/repair intermediates purified from wild type MMS treated cultures. **A.** 2D-gel analysis of replication/repair intermediates from wild type cells (JZ60) during a time course of incubation with 0.03% MMS. Times of MMS incubation are given below the panels. Two different restriction fragments from the rDNA region were analysed. The position of restriction sites and genetic elements of the rDNA region is outlined in the line drawing above the panels. **B.** Quantification of the ratio (%) of the intensity of repair intermediates and spike intermediates versus the intensity of the Y-arc for the panels shown in A. **C.** 2D-gel analysis of replication/repair intermediates from wild type cells (JZ60) treated for 1 h with increasing amount of MMS. **D.** Interpretation of replication/repair intermediates profiles from untreated or MMS-treated wild type cell cultures.

To determine the role of Swi1, Swi3 and Pol α in the alkylation damage response, we established an assay for detecting alkylation damage-specific repair intermediates in wild type cells. In *S. cerevisiae* alkylation repair intermediates have been detected using 2D-gel electrophoresis of replication intermediates isolated from MMS-treated cells [26], [27], [28], [29]. Since the migration pattern of these intermediates closely matched that of Holliday junctions formed by recombination they were suggested to be sister chromatid junctions formed behind the replication fork, due to the presence of single stranded regions created during replication of damaged template DNA. To detect alkylation specific intermediates in *S. pombe* we used genomic DNA digested either with *Hin*dIII/*Kpn*I or with *Bam*HI restriction enzymes, for the analysis of the fragment corresponding to rDNA repeat region, *ars3001*. We prepared two probes for this region, one of which covers the replication origin (the *Hin*dIII/*Kpn*I fragment), and another that covers the replication termination barrier (the *Bam*HI fragment). We determined the 2D-gel patterns at the *ars3001* locus in the presence and absence of MMS. We varied the length of drug exposure (Figure 3A) and drug concentrations (Figure 3C). At the *ars3001* replication origin, the profile of replication intermediates changed after 10 minutes following exposure to MMS (Figure 3A & B). A similar change was also observed at the terminator region albeit only after 30–60 minutes of incubation with MMS. At the replication origin region, the bubble-arc (typical for fragments containing the replication origin) was present in untreated cells but disappeared in MMS-treated cells. Interestingly at both the replication origin and the barrier regions a band appeared below the Y-arc only in the presence of MMS, suggesting the presence of an alkylation damage repair intermediate (Figure 3D). The amount of these intermediates increased with time (Figure 3A and B). Likewise, increasing the dose of MMS from 0.01% to 0.03% also lead to an increase in signal intensity, while no further increase was observed at 0.06% MMS (Figure 3C). We also observed a slight increase in the intensity of the spike signal constituted by Holliday junction intermediates, similar to what was observed for *S. cerevisiae* cells treated with MMS [26], [27], [28], [29] (Figure 3B).

### *swi1*, *swi3* and *swi7* mutations abolish the formation of alkylation damage repair intermediates

**Figure 4:**
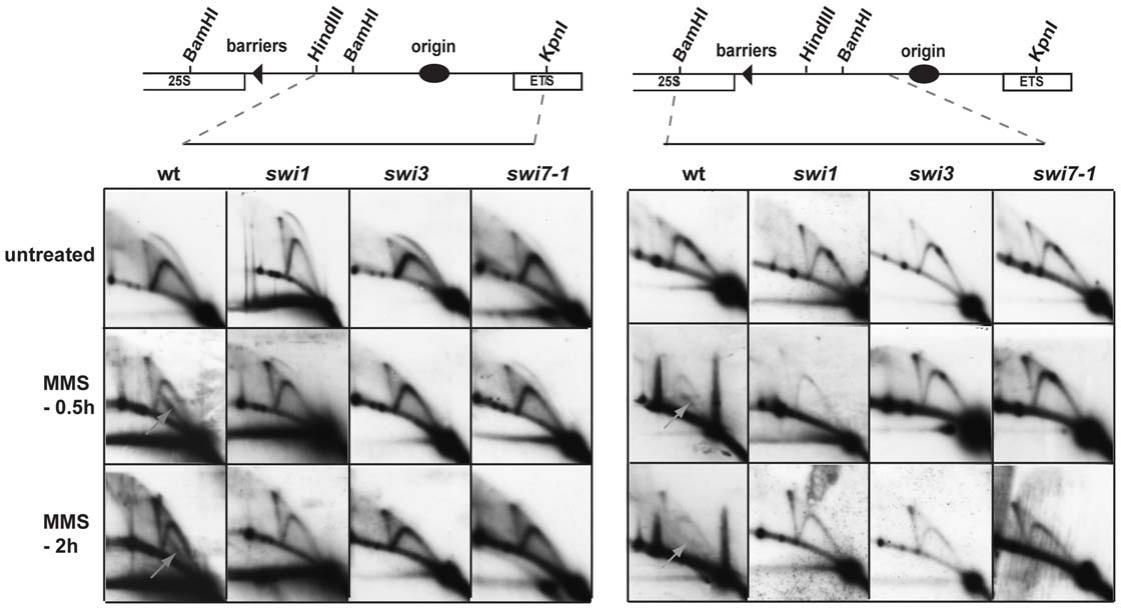
Analysis of replication/repair intermediates purified from wild type, *swi1*, *swi3* and *swi7-1* mutant yeast cells treated by MMS. 2D-gel analysis of replication/repair intermediates from wild type (JZ60), *swi1* (E111), *swi3* (E146) and *swi7-1* (JZ468) cells treated with MMS. The yeast genomic DNA was purified from cell cultures incubated without or in presence of 0.03% MMS for 0.5 and 2 h. The repair intermediates in the wild type panel are indicated with the gray arrows.

To determine whether Pol α, Swi3 and Swi1 are required for formation of alkylation damage repair intermediates, we performed 2D-gel analysis of the rDNA region from MMS-treated *swi1*, *swi3* and *swi7-1* mutant cells. While we detected the alkylation damage repair intermediates in wild-type cells after 0.5 and 2 h of MMS treatment, they were notably absent in *swi1*, *swi3* and *swi7-1* mutant cells. This was true for both the replication origin and termination barrier regions (Figure 4). Thus, Pol α, Swi3 and Swi1 are required for formation of alkylation damage repair intermediates.

### Replication coupled repair intermediates produced during alkylation damage are not generated by HU treatment

**Figure 5:**
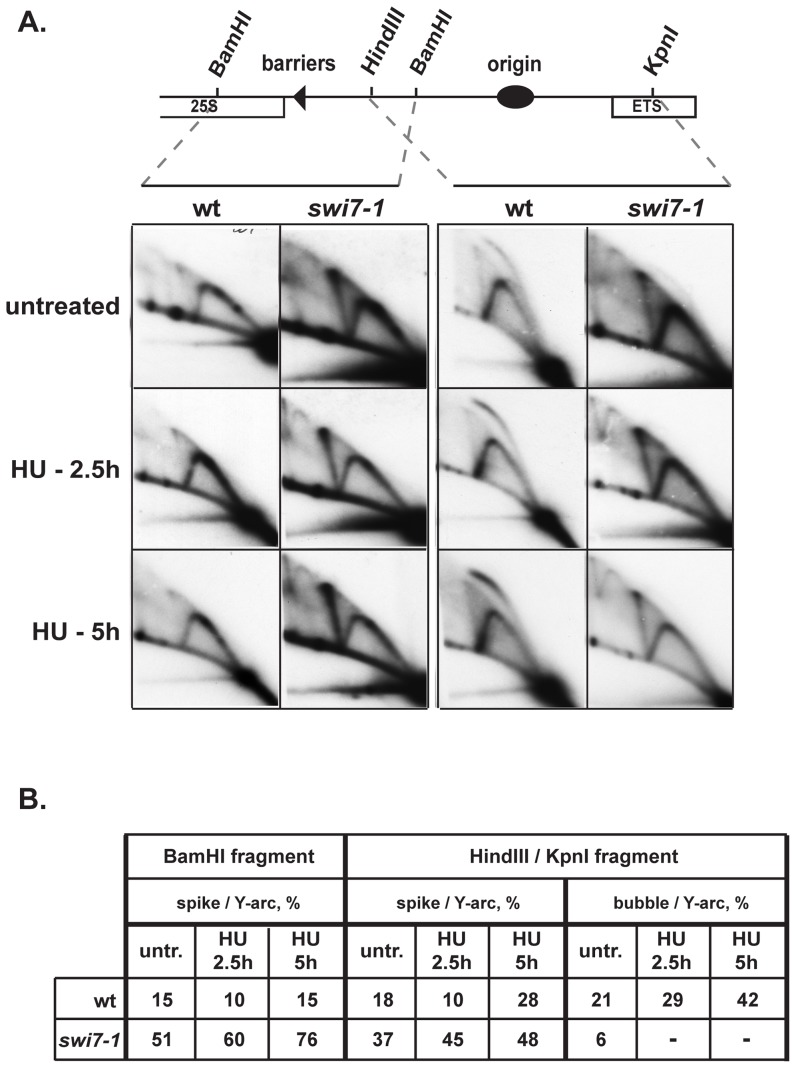
Analysis of replication/recombination intermediates purified from hydroxyurea treated wild type and *swi7-1* mutant yeast cells. **A.** 2D-gel analysis of replication/recombination intermediates from wild type cells (JZ60) and *swi7* (JZ468) during a time course of incubation with 12 mM HU. Times of HU incubation are given to the left of the panels. Two different restriction fragments from the rDNA region were analysed. The position of restriction sites and genetic elements of the rDNA region is outlined in the line drawing above the panels. **B.** Calculation in ratios (%) of bubble arcs (replication intermediates) and spikes (recombination intermediates) versus Y-arc intermediates.

We then tested whether the intermediates generated by MMS treatment were also generated by HU treatment. Earlier studies have shown that there is an increased instability of replication forks following HU treatment in *swi1* and *swi3* mutant strains [11], [12]. Importantly, 2D-gel analysis of replication intermediates for mutant cells suggested that the instability was due to single stranded regions forming behind the replication fork [11], [12]. We also wanted to test whether similar genetic instability could be observed for the *swi7-1* strain when cells were exposed to HU. Using 2D-gel analysis, we tested the replication intermediates purified from wild type and *swi7-1* mutant cell cultures, treated with HU for 2.5 and 5 h (Figure 5A). In replication/recombination intermediate profiles of wild type cells, obtained at the origin and barrier regions, we did not detect the intermediates that appear after MMS treatment. We observed that after HU treatment the ratios of bubble arc versus Y-arc increased for the wild-type stain (Figure 5A and B). Such an accumulation of bubble intermediates was previously observed [11]. At the same time in *swi7* mutant cells, the bubble arc was barely visible in the DNA sample from untreated cells and undetectable in HU-treated cells. This shows that the *swi7-1* mutation reduces the accumulation of bubble intermediates at *ARS3001*. Interestingly, a significant increase of the signal that corresponds to Holliday junction (HJ) intermediates was detected in *swi7-1* but not in wild type cells. This increase can be observed in untreated cells, and with a slightly increased intensity in HU treated cells (Figure 5A and B). Finally, we did not observe any signals below the Y-arc, as previously observed for *swi1* and *swi3* mutant cells exposed to HU, suggesting that no single stranded regions are forming behind the progressing replication fork [11], [12], [13]. Our data suggest that the *swi7-1* mutation causes genetic instability during DNA replication albeit different from that observed in *swi1* and *swi3* mutant strains.

### The *swi7-1* mutation causes genetic instability

**Figure 6:**
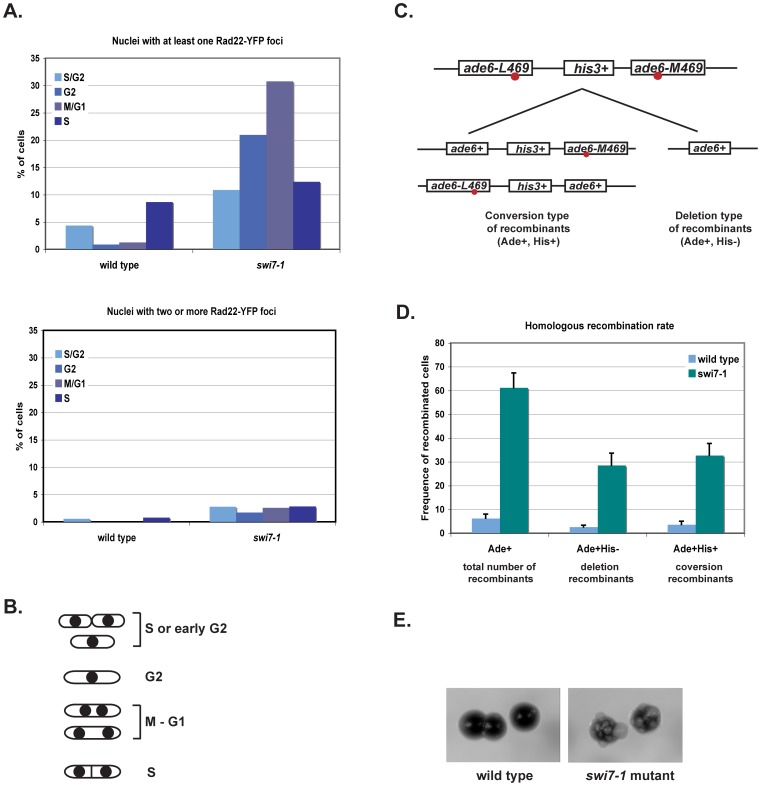
*swi7-1* mutation causes genetic instability. **A.** Quantification of Rad22-YFP foci in *swi7-1* mutant and wild-type cells. Wild type (ENY670) and *swi7* (MK140) mutant cells expressing Rad22-YFP were grown at room temperature until the mid-log phase. A small aliquot of cell culture was used for analysis by microscopy. About six hundred cells were analysed for each strain. **B.** Line drawing displaying the different cell morphologies through the cell cycle in fission yeast. **C.** Assay for quantification of recombination frequencies. Schematic drawing of the intrachromosomal recombination substrate and of the possible outcomes of recombination events. The red circles indicate the approximate positions of the mutations. **D.** Quantification of the recombination rate in wild-type (JZ518) and *swi7* (MK226) mutant strains. The histograms displays the recombination rates for the substrate shown in panel E. Rates of total number, conversion- and deletion-type recombination events are shown. The given values are the means of recombination frequencies for independent colonies. **E.** Visualization of recombination rates by growth on media containing limited adenine. *swi7* mutant displays increased sectoring on YE medium due to recombination at the direct repeats of *ade6* in the substrate. Cells with *ade6^−^* mutations in the intact substrate turn red on YE medium due to accumulation of a slightly toxic red pigment. Cells with the recombined wild type *ade6^+^* gene do not accumulate the pigment and are white (sectors in *swi7* colony).

To directly address whether the *swi7* mutation caused genetic instability as suggested by the increased presence of HJ-intermediates we used two approaches. To visualize DNA damage in living cells, we utilized cells that express Rad22-YFP from its endogenous promoter at its genomic locus. Rad22 is a homologue of budding yeast Rad52 [30] and was shown to bind to single-stranded DNA (ssDNA) during homologous recombination, leading to formation of Rad22-YFP DNA repair foci [31]. In comparison to wild type cells, we detected a highly elevated level of Rad22-YFP DNA repair foci in *swi7-1* cells (Figure 6A & B). The proportion of *swi7* cells with repair foci appears in G2, reaches its peak in M/G1 phase of the cell cycle and is maintained at significantly high levels throughout G1 and S phases. This observation indicates that the *swi7-1* mutant cells accumulate DNA damage after replication that is repaired mainly in the M/G1 phase of the cell cycle.

To test whether *swi7* cells have increased rates of homologous recombination (HR we used a recombination substrate that consists of a direct repeat of two *ade6^−^* heteroalleles separated by a wild-type *his3^+^* gene [32] (Figure 6C). The majority of HR occurs between sister chromatids, and these events are normally genetically silent. However, heteroallelic direct repeats on the same chromosome enable sister chromatid recombination to be monitored genetically. This is because unequal sister chromatid recombination between heteroalleles gives rise to genetically detectable recombinants. These can occur as deletions, in which one repeat and the intervening DNA are lost through either reciprocal crossing-over, or by non-reciprocal events such as single-strand annealing or break-induced replication. Deletion events recreate a wild type *ade6* ^+^ at the cost of the *his3* ^+^ gene and produce cells with an Ade+His^−^ phenotype. Recombination can also proceed as a gene conversion, which maintains both repeats and their intervening sequence. Gene conversions restore one fully active *ade6^+^* gene while leaving the *his3^+^* gene unaltered. Thus, gene conversions produce cells with an Ade^+^His^+^ phenotype. This recombination substrate was introduced into *swi7-1* background by a genetic cross. We observed much higher levels of both deletions and conversions in *swi7-1* mutant strain as compared to wild type cells (Figure 6D). The increased rate of mitotic recombination in mutant cells could also be observed on YE plates with limiting adenine. On YE medium, the two *ade6* mutations confer a red colour to the colony due to accumulation of a slightly toxic intermediate in the adenine biosynthesis pathway. Sectors, containing *ade6^+^* cells, appear larger and white due to reduced abundance of this toxic intermediate. The *swi7-1* mutants displayed an increased frequency of forming white sectors compared to the wild type strain, indicative of a higher recombination rate (Figure 6E).

### Pol α and Fen1 work in the same pathway in the alkylation damage response

To address if Pol α has a specific role in alkylation damage repair we analysed genetic interactions between *swi7-1* and four mutations namely *rad13*, *mag1*, *rev3 and rad2* that affect known repair pathways. *Rad13* encodes a DNA repair nuclease that acts in nucleotide excision repair (NER) [33] and has single-stranded DNA binding activity. Mag1 is a DNA-3-methyladenine glycosylase that initiates base excision repair (BER) [34]. Rev3 is the catalytic subunit of the B-family trans-lesion DNA polymerase Pol*ζ*, an enzyme which has the ability to bypass *cis-syn* thymine-thymine (T-T) dimers [35]. *Rad2* encodes a flap endonuclease-1 (Fen1), which is essential for both replication and repair. Fen1 is involved in Okazaki fragment processing during the lagging-strand DNA synthesis and in the long-patch BER pathway where it excises displaced oligonucleotides. It is also involved in resolution of Holliday junctions during homologous recombination [36].

**Figure 7:**
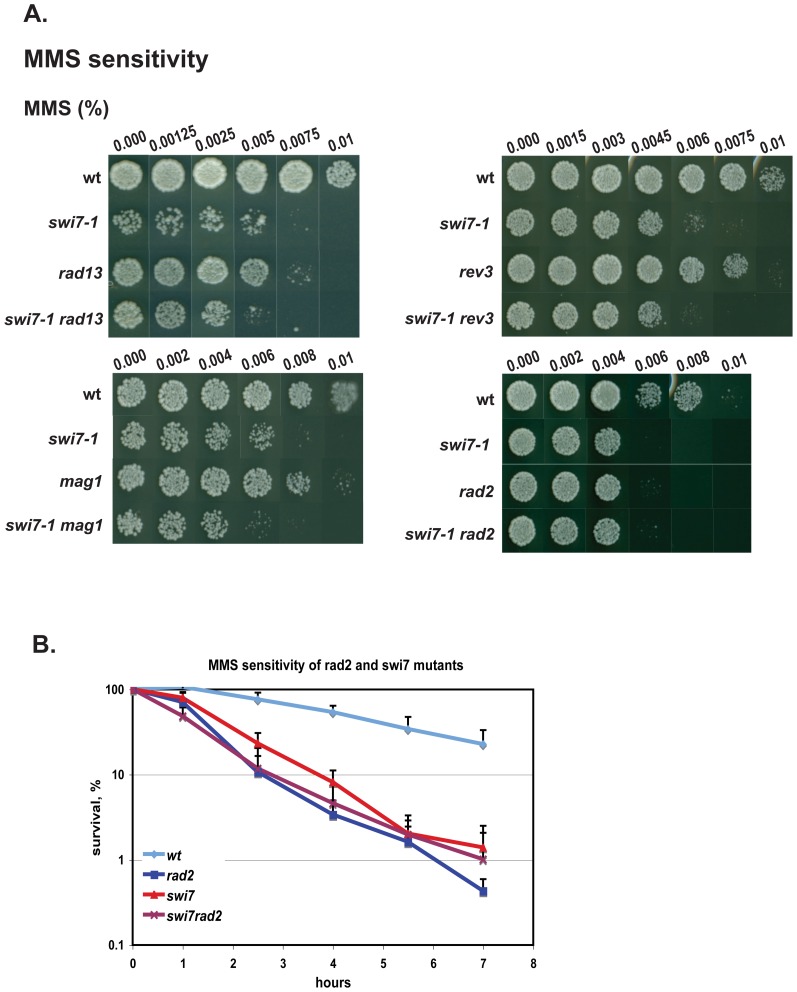
Genetic interactions of *S. pombe swi7-1* mutant in the response to DNA damage. **A, B** and **C.** Analysis of sensitivities of single and double mutant strains to increasing concentrations of MMS (**A**). Approximately 100 cells from mutant and wild type strains were plated on the medium, containing methyl methanesulfonate (MMS) or hydroxyurea (HU) at concentrations given above the columns, or were exposed to UV irradiation at the indicated intensities. The plates were incubated at 33°C until colonies were formed. Strain names are as follows *wt* (JZ60), *swi7-1* (JZ487 or JZ468), *rad13* (SV66), *mag1* (AM006), *rev3* (SV54), *rad2* (SV59), *swi7rad13* (MK130), *swi7mag1* (MK175), *swi7-1 rev3* (MK190), *swi7-1 rad2* (MK125). **B.** Survival of *wt*, *swi7-1*, *rad2* and *swi7-1 rad2* strains exposed to MMS. Logarithmically growing cultures were incubated in the presence or absence of 0.03% MMS. Samples were taken at indicated time points, and dilution series of cells for each culture were plated on YEA media and incubated at 33°C until colonies formed. The survival is shown as the relative percentage compared to the values obtained using the untreated starting cultures.

We generated double mutants of *swi7-1* with *rad13*, *mag1*, *polζ* and *rad2* deletions by genetic crosses and compared the MMS sensitivities of single and various double mutant combinations. *swi7-1* in combination with *rad13*, *mag1* and *polζ* mutations had an additive effect on MMS sensitivity (Figure 7A), demonstrating that Pol α and Rad13/Mag1/*polζ* have distinct functions in the alkylation damage response pathway. Thus the *swi7-1* mutation does not uncover a role of Pol α in either NER or BER repair pathways or directly in post-replicative trans-lesion synthesis. In contrast, s*wi7-1* in combination with *rad2* had no additive effect on MMS sensitivity (Figure 7A) in both the chronic exposure assay and the short-term exposure assay (Figure 7B). These data suggest that Polα and Fen1 work in the same pathway in the alkylation damage response.

### Fen1 is required for formation of alkylation damage-specific repair intermediates

**Figure 8:**
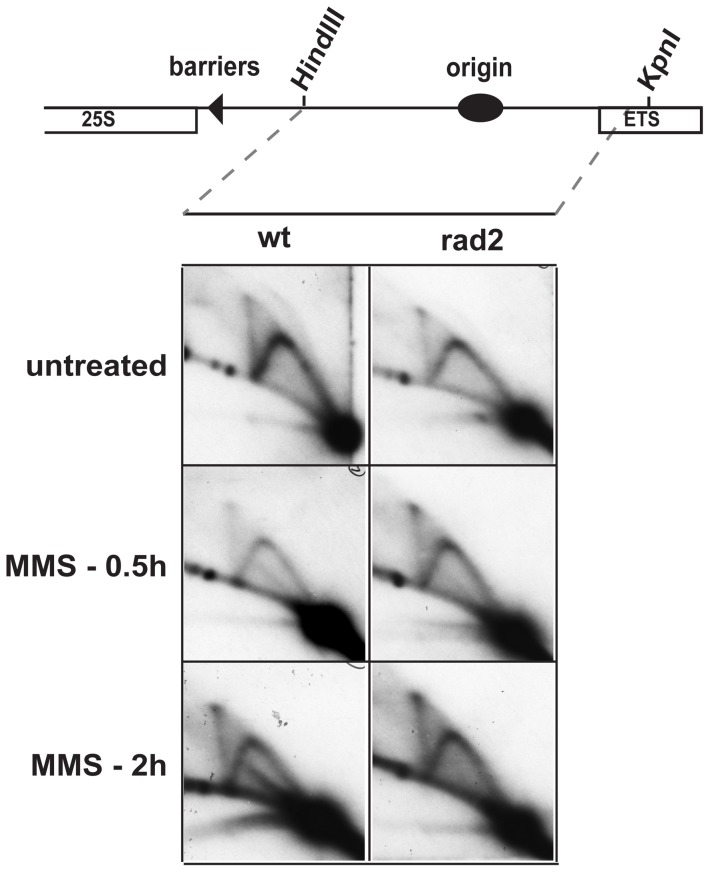
The *rad2* mutation abolishes the presence of the novel alkylation damage repair intermediates. 2D-gel analysis of untreated and MMS treated wild-type and *rad2* strains. The *Hin*dIII/*Kpn*I fragment of the rDNA was analyzed in the experiment, see Figure 2 line drawing. Cells were treated with 0.03% MMS for 0.5 and 2 h as shown.

Since MMS sensitivity data suggest that Pol α and Fen1 work in the same pathway in the alkylation damage response we investigated whether Fen1 is required for alkylation damage-specific repair intermediates like Pol α. The alkylation damage-specific intermediate consistently observed in wild type strain was strikingly absent from the *rad2* strain (Figure 8). These data suggest that Swi1, Swi3, Pol α and Fen1 act together in the alkylation damage repair pathway.

### Swi1, Swi3 and Fen1 might have distinct functions during alkylation damage response

**Figure 9:**
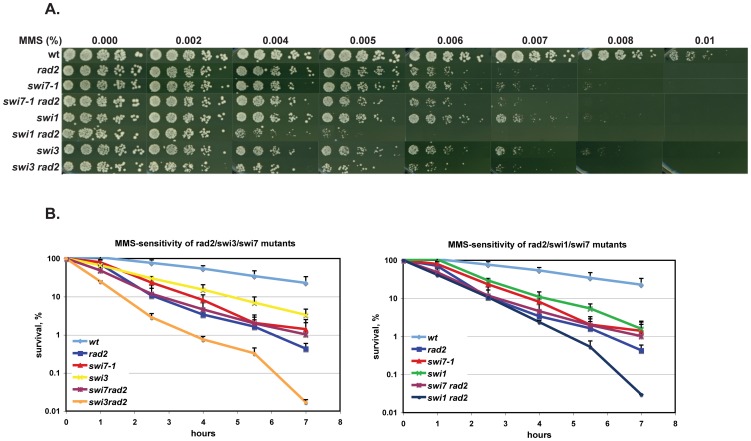
*Swi1* and *Swi3* possess additional roles in the alkylation damage response. Analysis of sensitivities of wild-type (JZ60), single *swi7-1* (JZ468), *rad2* (SV59), *swi1* (E111), *swi3* (E146) and double mutant *swi7-1 rad2* (MK125), *swi1 rad13* (MK239), *swi3 rad2* (MK243) strains to increasing concentrations of MMS or hydroxyurea (HU). Approximately 100 cells from mutant and wild type strains were plated on the medium, containing methyl methanesulfonate (MMS) or hydroxyurea (HU) at concentrations given above the columns. The plates were incubated at 33°C until colonies were formed. **B.** Survival curves for yeast cells exposed to MMS. Logarithmically growing cultures were treated with 0.03% MMS, samples were taken at indicated time points, and dilution series of cells for each culture were plated on YEA media and incubated at 33°C until colonies formed. Strain names are given in A.

*swi1*, *swi3* and *swi7-1* mutations abolish *mat1* imprinting, and all three mutations cause a hypersensitivity to MMS, HU and UV. However, it is not known whether these proteins act together with Fen1 during the alkylation damage response. We therefore analyzed genetic interactions between *swi1*/s*wi3* mutations and *rad2* under DNA damaging conditions. In the chronic exposure assay (Figure 9A), both *swi1* and *swi3* mutations in combination with *rad2* displayed additive effect on MMS sensitivity. This was particularly pronounced for *swi1* compared to *swi3*. Similar additive effects between *swi1/swi3* and *rad2* were observed in the short-term survival assay (Figure 9B). These data suggest that although alkylation–specific DNA intermediate fail to accumulate in *swi1*, *swi3* and *rad2* strains, Swi1, Swi3 and Fen1 might have distinct functions during the alkylation damage response.

## Discussion

In this study we addressed whether the *swi7-1* allele that abolishes imprinting at the *mat1* locus confers any other defects outside the *mat1* locus. Importantly, we show that the *swi7-1* mutation confers sensitivity to DNA damaging agents MMS, HU and UV (Figure 1). The level of sensitivity is similar to what previously has been observed for *swi1* and *swi3* mutant cells [11], [12], [13]. One question is whether the observed sensitivity is due to a partial loss of Pol α catalytic activity or whether it is due to loss of a more specialized function of Pol α conferred by the residue mutated in the *swi7-1* allele. Our data does establish that the unperturbed DNA replication process is affected by *swi7-1* mutation. We observed an increased level of intermediates with an mobility similar to HJs in *swi7-1* strains by 2D gel analysis (Figure 4 & 5). Increased levels of HJs have previously been observed in a *S. cerevisiae* Pol α mutant [37]. We also observed an increased proportion of cells with a “cut” phenotype, as well as an increased level of Rad22-YFP repair foci with a peak in M/G1, suggesting that the *swi7-1* mutation causes DNA damage that is mostly repaired in M-phase (Figure 2 and 6). Finally, we observed an increased level of homologous recombination in *swi7-1* cells (Figure 6). All these data show that *swi7-1* mutation affects the general replication process without affecting cell viability. It is noteworthy that mutations in several replication-associated enzymes like DNA polymerases, ligases, topoisomerases and DNA helicases lead to increased mitotic recombination (for reviews see [38], [39])

The genetic instability observed in the *swi7-1* strain might also explain why Pol **α** earlier was shown to regulate chromatin organization at the mating-type locus, centromeric and telomeric regions. In that study, the *swi7-1* mutation was shown to affect the physical interaction between Pol α and heterochromatin protein Swi6 [40]. However, there is no evidence that links loss of *swi6* function with effects on either the DNA replication or *mat1* imprinting. Potentially, this suggests that the *swi7-1* point mutation affects several different functional interactions. Alternative explanation is that the loss of silencing observed in the *swi7-1* background could be due to defects during replication leading to DNA damage repair processes acting at the silenced regions.

Although the observed DNA damage sensitivity or the genetic instability we observed in the *swi7-1* strain could be partly due to general lowering of the Pol α catalytic function, a specific role of Pol α in alkylation damage repair might exist as the *swi7-1* mutation abolishes alkylation damage induced repair intermediates. Crucially genetic data suggest that Pol α and Fen1 work in the same pathway in alkylation damage repair.

**Figure 10:**
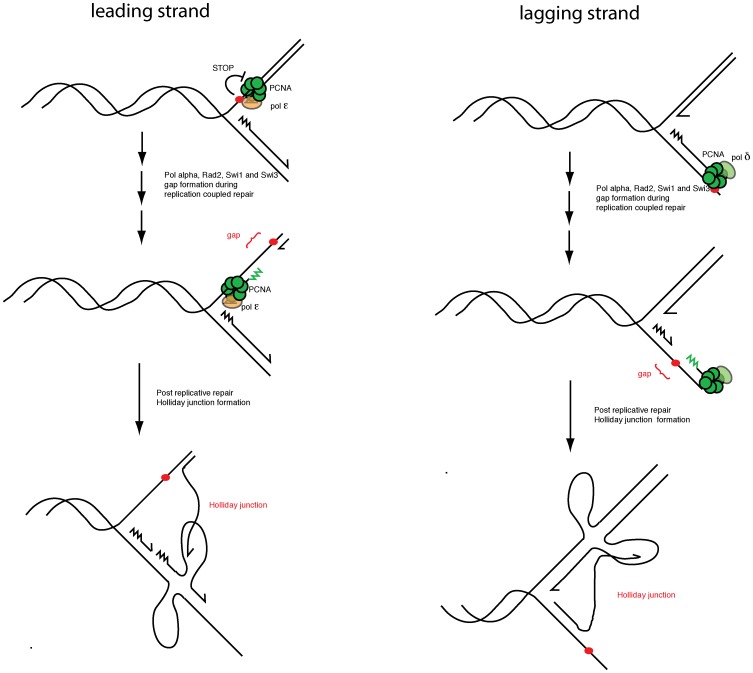
Model figure. Alkylation damage on the leading (left half) and lagging (right half) strand template is repaired by a *rad2*, *swi1*, *swi3* and *swi7-1* dependent pathway leading to the formation of single-stranded regions behind but in close proximity to the progressing fork. These intermediates are detected by 2D-gel analysis below the y-arc for the wild-type strain (Figure 2). The single-stranded regions lead to Holliday junction (HJ) formation either by formation of double-stranded breaks or by fork regression.

It is possible to envisage how some of the genome wide defects observed in the *swi7-1* strain could be explained by its loss of the imprinting function. We have earlier proposed that ribonucleotides similar to the *mat1* imprint are introduced at stalled forks at other genomic regions, the difference being that outside *mat1* such ribonucleotides would be excised in the same cell cycle [41]. Our work at the *mat1* imprint has shown that there are no sequence requirements other than those required for replication pausing and maintenance of the ribonucleotide imprint [42]. While the *mat1*imprint is shielded away from the DNA repair machinery by unknown mechanisms, imprints at other genomic regions could mark sites of DNA damage for recruitment of repair pathways. Pol α has a potential role in introducing these imprints which is affected by the *swi7-1* mutation. This will explain why the *swi7-1* mutants are defective in diverse processes like imprinting, intra-S checkpoint signalling and alkylation damage response. Such a repair pathway could be especially important in *S. pombe* that is predominantly haploid. *S. pombe* cells are unable to repair DNA damage by recombination with a homologous chromosome. The G1 cell-cycle in *S. pombe* is very short, followed by a short S-phase but a much longer G2 phase. The extended G2 phase might have evolved to permit DNA damage repair through recombination with the sister chromatid. If DNA is damaged in S-phase, the cells potentially could repair the damage in front of the replication fork. This could lead to cell death due to stalled or collapsed replication forks as repair can involve strand breakage. Alternatively the cells could replicate through damaged DNA and rely on post-replicative repair behind the replication fork in S or G2 (Figure 10). Thus, imprints like the *mat1* imprint could play a crucial role for marking DNA damage for post-replicative repair when the replication fork is stalled at sites of DNA damage. Here, Swi1 and Swi3 could act to pause the replication fork. We do know that the replication fork pause *MPS1* is dependent on Swi1 and Swi3 function, and that Swi1 and Swi3 are thought to have a general role at stalled replication forks [9], [11], [12], [13]. In addition, we previously showed that Swi1 and Swi3 are part of the intra-S phase alkylation damage response pathway, possibly acting to stabilize replication forks stalled at damaged bases [13].

Interestingly, we detect alkylation-damage induced repair intermediates in wild-type cells that are absent from *rad2*, *swi1*, *swi3* and *swi7-1* strains (Figures 3 and 4). This strongly supports the notion that these four genes are directly involved in repair of alkylation damage. The intermediates run as a line below the arc of Y-shaped intermediates on 2D-gels, suggesting that large single-stranded regions and/or breaks are formed behind the replication fork in one of the arms of the Y-structured intermediates (Figure 3D & Figure 10). Interestingly, these putative alkylation damage intermediates differ from those observed in MMS-treated *S. cerevisiae* cells where spike signals migrating at the same position as HJ are observed [26], [27], [28], [29]. In *S. cerevisiae* it has been shown that the appearance of these spike signals depends on Rhp51 [27], [28]. While, we also observed an increase in the spike signal MMS-treated *S. pombe* cells, the increase was not as large as that observed in *S. cerevisiae*.

Also, the repair intermediates below the Y-arc are only observed when cells are treated with MMS and not when they are exposed to HU (Figure 3 & 5), suggesting that it is not per se the stalling of forks that leads to their formation, but a specific response to MMS damage. In this context it is interesting that while the *swi7-1* mutation had additive effects with *rad13*, *mag1*, or *rev3*, in terms of MMS sensitivity, it does not with *rad2* (Figure 7). Since *rad2*/Fen1 has been implicated in long patch BER, it is possible that imprints made by Pol α could play a role in long patch BER. Alkylation-specific repair intermediates are also absent in MMS-treated *rad2* cells, we suggest that Fen1, Swi1, Swi3 and Pol α act together in a DNA damage repair pathway (Figure 4, 7, 8 and 9). Here it is important to note that Fen1 is a nuclease that can recognize and cleave at single as well as multiple ribonucleotides inserted into DNA [43].

Similarly, the observation that the *swi1* and *swi3* have additive effects when combined with *rad2*, while all three mutations abolish the presence of the novel alkylation damage repair intermediates (Figures 3 and 8) can be explained. Firstly, it should be noted that in the chronic exposure assay the additive effect observed when *rad2* and *swi3* are combined is very minor (Figure 9). Secondly, Swi1 and Swi3 might have more general functions in the response to alkylation damage. Potentially, this could be through their involvement in the intra-S phase checkpoint response [11], [12], [13]. Interestingly, we observe that *swi7-1* also affects the intra-S phase checkpoint response to MMS exposure, but not the S-M checkpoint (Figure 2). Alternatively, the absence of alkylation damage repair intermediates in the *swi7-1* background could indirectly affect the cells' ability to arrest the cell-cycle in response to alkylation damage.

In summary, our data suggest that Swi1, Swi3, Pol α and Fen1 act together during alkylation damage repair response. Determining how these evolutionarily conserved proteins precisely work at the sites of DNA damage is a key challenge for the future.

## Materials and Methods

### *S. pombe* strains

The following strains were used for the study: **AM006** (*h^90^, leu1-32, Δ mag1*::*ura4*) [44]; **E111** (*h^90^, ade6-210, leu1-32, his2, swi1-111*) [10]; **E146** (*h^90^, ade6-210, leu1-32, his2, swi3-146*) [10]; **ENY670** (*h^−^, leu1-32, ura4-D18, his3-D1, rad22-YFP::Kan^R^*) [11]; **JZ60** (*h^90^, ade6-210, leu1-32*); **JZ468** (*h^90^, ade6-210, leu1-32, swi7-1*); **JZ473** (*h^90^, ade6-210, 1.5::LEU2, Δchk1::ura4^+^*); **JZ474** (*h^90^, ade6-210, 1.5::LEU2, Δrad3::ura4^+^*); **JZ475** (*h^90^, ade6-210, 1.5::LEU2, Δ cds1::ura4^+^*); **JZ487** (*h^90^, ade6-210, leu1-32, Δura4, swi7-1*); **JZ518** (*h^−^, ade6-M365::pUC8/h1936, ade6-L469, leu1-32, ura4-D18, his3-D1*) [45]; **MK125** (*h^90^, leu1-32, ura4-D18, swi7-1, Δrad2::ura4*); **MK130** (*h^90^, leu1-32, ura4-D18, swi7-1, Δrad13::ura4*); **MK140** (*h^90^, leu1-32, swi7-1, rad22-YFP::Kan^R^*); **MK175** (*h^90^, leu1-32, ura4-D18, swi7-1, Δmag1::ura4*); **MK190** (*h^90^, leu1-32, swi7-1, Δ rev3::Kan^R^*); **MK226** (*h^90^, ade6-M365::pUC8/h1936, ade6-L469, leu1-32, his3-D1, swi7-1*); **MK239** (*h^90^, ade6-210, leu1-32, ura4-D18, swi1-111, Δrad2::ura4*); **MK243** (*h^90^, ade6-216, leu1-32, ura4-D18, swi3-146, Δrad2::ura4*); **SP246** (*h^90^, leu1-32, ura4-D18, pol1-1*) [46]; **SP262** (*h^90^, leu1-32, pol1-H4*) [47]; **SV54** (*h^90^, ade6-210, leu1-32, ura4-D18, Δ rev3::Kan^R^*); **SV59** (*h^90^, ade6-210, leu1-32, ura4-D18, Δrad2::ura4*); **SV66** (*h^90^, ade6-210, leu1-32, ura4-D18, Δrad13::ura4*); **SV232** (*h^90^, ade6-210, leu1-32, ura4-D18, pol1-13ts*).

### General techniques

The methods used for genetic analyses of fission yeast have been described previously [48], [49], [50]. Strains were grown in yeast extract (YE) medium supplemented with 225 mg/l of adenine (YEA medium), in YE5S medium, containing 250 mg/l of 5 amino acids (histidine, lysine, leucine, uracil, adenine), in AA-Media, containing Bacto Yeast nitrogen base added dropout amino acids mixes (types: -leu, -ura, -ade, -his), or in PMA+ medium.

### Quantitative tests for sensitivity to chemical treatments and UV irradiation

For the chronic exposure assays, serial 5-fold dilutions of each strain (starting from cultures containing 0.2×10^6^ cells/ml) were prepared in 96-well microliter plates and plated with a 48-prong replicator (Sigma) onto YEA media containing the given concentrations of methyl methansulfonate (MMS) or hydroxyurea (HU). For the UV irradiation sensitivity test, cells were plated on YEA plates and irradiated using a Stratagene UV Crosslinker with the given energy. For short-term survival assays, yeast cultures (100 ml) were incubated at 33°C until they reached logarithmical growth and either MMS (0.03%) or HU (12 mM) were added. Serial 10-fold dilutions of each strain (starting from ∼5000 cells/ml) were prepared in 1 ml YEA medium using culture aliquot taken at different time points. Dilution aliquots were plated on YEA medium (3 repeats each), and incubated at 33°C until colonies were formed. Colonies were counted, and the time course of cell survival under drug treatment was calculated as the survival percentage of the wild type strain.

### 2D-gel electrophoresis

Cells grown in YEA media at 33°C were arrested with 0.1% sodium azide and chilled by mixing cultures with equal volume of ice in a centrifuge bottle. Approximately 2–4×10^9^ cells were harvested, washed in cold water and frozen at −70°C. Genomic DNA was purified as described elsewhere [51], [52]. For analysis of *ars3001*, 10 µg of DNA was digested with either *Hin*dIII/*Kpn*I, or *Bam*HI (overnight, 37°C). The volume and the concentration of NaCl were increased to obtain 1 ml of DNA solution containing 1 M NaCl and purified on Benzoylated Naphthoylated DEAE-Cellulose (BND-cellulose, Sigma) for enrichment of replication intermediates. Two dimensional (2D) electrophoresis was performed as previously described [53]. First dimension electrophoresis was run overnight at 1.5 V/cm. After electrophoresis, gels were photographed with long-wave UV light, the lanes containing the DNAs of interest were cut out, placed at the top of empty 24×12 cm gel tray, and then embedded in 1.2% agarose. Second-dimension electrophoresis was run in TBE buffer, containing 0.3 µg/ml of ethidium bromide for several hours at 7.5 V/cm, 4°C. Southern analysis was done as described elsewhere. The *ars3001* (for *S. pombe* rDNA repeat) radioactive probes were prepared using as templates the 50 ng of PCR products obtained from *S. pombe* genomic DNA with one of two pairs of primers (for the *Hin*dIII/Kpn1 fragment: forward- AGTACAAACAAACCAAGCGTGTTA, reverse- TCTTTCCAATGCACCGCGATGCCAACACA, for the *Bam*HI fragment: forward- GGGATAAGGATTGGCTCTAAGG, reverse- CAATCCATCCATCCATCAAACTTTATAC).

### Monitoring of homologous recombination

The method is described in detail in [32]. To test whether the recombination substrate was intact the strains were streaked from their stocks onto YE medium, containing low (10 mg/L) adenine and without histidine supplement (YE/low ade, his^−^ plates), and then after several days of incubation at 30°C, replica plated onto YE/low ade and YEA media. YEA plates allowed us to calculate the total number of colonies and YE/low ade served to determine the status of the *ade6* substrate. For each strain fifteen colonies, which were red on the YE/low ade plate, were taken from YEA plates and suspended in 1 mL of water. This neat cell suspension was used to prepare 10^−3^ dilution series. An aliquot, 100 µL, of each neat suspension was spread on YE medium, supplemented with 200 mg/L of guanine to select for ade^+^ colonies and 50 µL of each 10^−3^ dilution were spread onto YE/low ade plates. After several days of incubation at 30°C, colonies grown on YE/guanine plates were replicated onto AA/low ade, his^−^ plates and allowed to grow. Colonies on AA/low ade plates represented total amount of viable cells, colonies on YE/guanine plates were used to count Ade^+^ recombinant cells, and the growth on YE/low ade, his^−^ plates enabled us to distinguish between conversion, Ade^+^ His^+^, and deletion, Ade^+^ His^−^, types of recombination. Colonies were counted and the obtained data were summarized in a table and plotted using the Microsoft Excel program.

### Detection of Rad22-YFP DNA repair foci

The protocol and the parental strain used are described in detail by [31]. Cells expressing Rad22-YFP were grown overnight at RT, diluted in 10 ml of YEA media and incubated at RT until they reached the mid-log phase. After incubation, cells were spun down, washed with PBS and suspended in a small amount of liquid. Five microlitres of suspension were dropped onto the slide and analysed using the API Deltavision RT system 2 and the filter for YFP.

### Flow cytometry analysis

Detailed method for fission yeast is described in [54]. For flow cytometry analysis, cells were fixed in 70% ethanol, pelleted, washed in 50 mM sodium citrate (pH 7.0), and incubated for at least 6 h (at 37°C) or overnight (RT) in 50 mM sodium citrate containing 0.1 mg/ml of RNase A. After sonication, cells were stained with 1 µM Sytox Green (Molecular Probes). The analysis was performed on Becton-Dickinson FACS Calibur flow cytometer. FlowJo software (Treestar, Inc.) was used to generate the histograms.

### Septation and binucleated-cells index

We used the protocol described in [55]. Cell samples were prepared as for FACS analysys using ethanol fixation. Approximately 50 µL of fixed cells were rehydrated in 1 mL water, vortexed and spun down. Water was removed and cells were suspended in a small amount of liquid. Five microliters of suspension were dropped onto slide and cells were fixed by heat at 70°C for several minutes. For nuclear and septum staining, 5 µL of mounting solution (50% glycerol, 1 mg/ml p-Phenylenediamine, antifade) containing DAPI (1 µg/ml) and Calcofluor (200 µg/ml) were added. All compounds were from Sigma. Septa and nuclei were visualized by fluorescence microscopy under ultraviolet light wavelength. Five hundred or more cells were analyzed for each sample.
